# Application of ARID1A to murine formalin-fixed paraffin embedded tissue using immunohistochemistry

**DOI:** 10.12688/f1000research.5514.2

**Published:** 2015-01-06

**Authors:** Will Howat, Jodi Miller, Ioannis Gounaris

**Affiliations:** 1 Cancer Research UK Cambridge Institute, University of Cambridge, Li Ka Shing Centre, Cambridge, CB2 0RE, UK

**Keywords:** immunohistochemistry, antibody, ARID1A, tissue

## Abstract

ARID1A is a known suppressor of tumour formation and the Human Protein Atlas antibody HPA005456 has been demonstrated in previous literature to stain tumour tissue by immunohistochemistry (IHC) in formalin-fixed paraffin embedded human tissue and human cell lines. This article details the validation of this antibody for immunohistochemistry of formalin-fixed paraffin embedded murine tissue using a Leica BondMax immunostainer. Using Western blot and IHC on murine wild-type and knockout tissue we have demonstrated that this antibody to ARID1A correctly stains murine tissue by immunohistochemistry.

## Introduction

ARID1A (AT-rich interactive domain 1a) is a member of the SWI/SNF family and its loss has been implicated as a factor in multiple premalignant and malignant conditions, including Barrett’s oesophagus and oesophageal carcinoma as well as endometrial and clear cell ovarian carcinomas and their precursor endometriotic lesions ^1– 4^. The ARID1A antibody from Human Protein Atlas is a rabbit antibody generated against a PrEST (Protein Epitope Signature Tag) fragment of the ARID1A gene and affinity purified against the same fragment ^5^. It is thus designated as being “mono-specific” in that the affinity purification removes any non-specific or low affinity binders to the peptide. Through the Human Protein Atlas, the antibody has been tested on a wide variety of human tissue types and human malignancies, as well as for expression in immunofluorescence on U-2 OS, A-431 and U-251 MG cell lines. This demonstrates a nuclear expression in all cell lines and in the majority of tissue types ^6^. However, the Western blot data were not supportive and did not produce staining corresponding to the expected size, although the data from a protein array did confirm a peak at the expected size. The antibody has been used to stain human colorectal cancers ^7^, clear cell carcinomas ^8^ and on a variety of clear cell cancer cell lines ^9^ by immunohistochemistry.

To our knowledge, whilst the sequence homology between mouse and human ARID1A is 95%, this antibody has not been qualified using knockout tissue and has not been tested or published on murine tissue and this work represents the first data to do so.

## Materials and methods

### Reagent details

Details of all reagents with reference to the immunohistochemical staining procedure can be found in Table 1.

**Table 1.** Details of ancillary reagents for immunohistochemistry.

Process	Reagent	Manufacturer	Catalogue Number	Concentration
Fixation	Neutral Buffered Formaldehyde	Sigma	HT501128	10%
Pretreatment	ER1 (Sodium Citrate, pH6)	Leica Biosystems	AR9961	Proprietary
	ER2 (Tris/EDTA, pH9)	Leica Biosystems	AR9640	Proprietary
	Enzyme 1 (Proteinase K)	Leica Biosystems	AR9551	100 µg/ml
Staining	Peroxide Block	Leica Biosystems	DS9263	Proprietary
	Streptavidin – HRP	Leica Biosystems	DS9263	Proprietary
	Diaminobenizidine (DAB)	Leica Biosystems	DS9263	Proprietary
	Haematoxylin	Leica Biosystems	DS9263	Proprietary
	DAB Enhancer	Leica Biosystems	AR9452	Proprietary
Washes/Blocks	Bond Wash (Tris Buffer)	Leica Biosystems	AR9590	Proprietary
	Antibody Diluent	Leica Biosystems	AR9352	Proprietary
	Avidin/Biotin Block	Vector Laboratories	SP-2001	Proprietary

### Antibody details

Anti-ARID1A is a monospecific rabbit polyclonal generated to a PrEST sequence – PGLGNVAMGPRQHYPYGGPYDRVRTEPGIGPEGNMSTGAPQPNLMPSNPDSGMYSPSRYPPQQQQQQQQRHDSYGNQFSTQGTPSGSPFPSQQTTMYQQQQQNYK ( Table 2). The homology of the PrEST sequence used as immunogen is 95%, when verified with BLAST against the mouse sequence. The lot number used for all validations was A40072 and for subsequent staining was D81856. A concentration of 1 µg/ml was used for initial validations and 0.5 µg/ml for final runs.

**Table 2.** Details of primary and secondary antibodies.

Antibody	Manufacturer	Catalogue Number	RRID
ARID1A	Atlas Antibodies	HPA005456	AB_1078205
GAPDH (Clone 14C10)	Cell Signaling	2118	AB_561053
Donkey anti-Rabbit Biotin	Jackson Immunoresearch	711-065-152	AB_2333077
Goat anti-Rabbit IRDye 680LT	Li-Cor Biosciences	926-68021	AB_10706309
Goat anti-Rabbit IRDye 800CW	Li-Cor Biosciences	926-32213	AB_621848

Donkey anti-rabbit biotin (Jackson Immunoresearch, Table 2) is specific for Rabbit IgG (Heavy and Light chains) and was affinity purified to remove cross-reactions to Bovine, Chicken, Goat, Guinea Pig, Syrian Hamster, Horse, Human, Mouse, Rat and Sheep. All slides were stained with a concentration of 4.8 µg/ml.

Anti-GAPDH was used as a loading control for Western blots and was a rabbit monoclonal (Cell Signaling, Table 2). Detection antibody for the Western blot for ARID1a was Goat anti-rabbit IR Dye 680LT (Li-Cor Biosciences, Table 2) used at a concentration of 0.1 µg/ml and detection of GAPDH was Goat anti-rabbit IR Dye 800CW (Li-Cor Biosciences, Table 2).

### Tissue details

All tissues and cell pellets used during the validation were fixed in Neutral Buffered Formaldehyde as specified ( Table 3) before being transferred directly to 70% ethanol for no longer than 3 days. Tissue processing was conducted on a Leica ASP300 through graded ethanols before clearing in xylene and impregnation in molten paraffin wax (Fisher). All tissue sections were cut on a Leica rotary microtome at 3 µm.

Arid 1a ^-/-^ mice were created by crossing Floxed *Arid1a* mice with *ROSA26 Cre-ERT2* mice and resultant genotyping. Loss of Arid1a expression is expected following intraperitoneal injection of Tamoxifen.

Floxed *Arid1a* mice were a gift from Dr. Peri Tate, Sanger Institute, Hinxton UK; *ROSA26 Cre-ERT2* mice were a gift from Prof Chambon, IGBMC, France. ES-2 cells were purchased from ATCC and RMG-II were a gift from Prof Huntsman, British Columbia Cancer Agency, Vancouver, Canada.

**Table 3.** Details of tissue and cell pellet used during the validation.

Species	Tissue Type	Strain/Cell line	Details	Fixation Time
Murine	Uterus	C57Bl6	Female	16 hrs
Murine	Uterus	C57Bl6 KO *Arid1a ^fl/fl^*	Female	24 hrs
Human	Cell Pellet	ES-2		20 hrs
Human	Cell Pellet	RMG-II		20 hrs

### Experiment details

***Western blot.*** Protein was extracted from the two clear cell carcinoma cell lines using a Tris-EDTA lysis buffer and run on a non-denaturing 3–8% Tris-acetate gel (Life Technologies). Following electrophoresis, the transfer membrane was probed with 0.2 µg/ml of anti-rabbit ARID1A (HPA005456) at 4°C overnight and 0.1 µg/ml anti-GAPDH (14C10) for the same length of time. Detection of the anti-rabbit ARID1A was with Goat anti-rabbit IRDye 680LT (Li-Cor Biosciences) and the GAPDH was with Goat anti-rabbit IRDye 800CW (Li-Cor Biosciences) both at 0.1 µg/ml.

***Immunohistochemistry.*** Slides were deparaffinised and rehydrated on a Leica ST5020 using Xylene (Sigma) for 2 × 10 mins and ethanol (Fisher), 2 × 100% ethanol followed by 1 × 70% ethanol for 5 mins each. Following staining, all slides were dehydrated, cleared and mounted and coverslipped in DPX (Fisher).

The antibody was validated on a Leica BondMax instrument using a Leica Intense R kit to a standardised in-house protocol. All reagents were from Leica as part of the Intense R kit and were conducted at room temperature, unless otherwise specified. All staining steps included individual washes in Leica Bond Wash after each step, as part of the protocol ( Table 4). A full protocol for the validated conditions can be found in the supplementary material. In this protocol, the step named “primary” refers to the anti-ARID1a primary antibody.

**Table 4.** Staining protocol for ARID1A immunohistochemistry.

Protocol steps	Reagent	Time (mins)
Antigen Retrieval	ER1	20
	Or ER2	20
	Or Enz1	10
Staining	Peroxide Block	5
	Avidin	10
	Biotin	10
	ARID1A	15
	Donkey anti-rabbit Biotin	8
	SA-HRP	8
	DAB	5
	DAB Enhancer	10
Counterstaining	Haematoxylin	5

A slide using the same conditions and retrieval but omitting the primary antibody was used to control for any background staining due to the retrieval and detection steps.

***Imaging.*** All slides were digitised using a Leica Scanscope AT2 at 0.5 µm/pixel resolution. Datasets can be viewed by downloading the Leica Imagescope free viewer at http://www.leicabiosystems.com/pathology-imaging/aperio-epathology/integrate/imagescope/.

## Results

**Figure 1.**
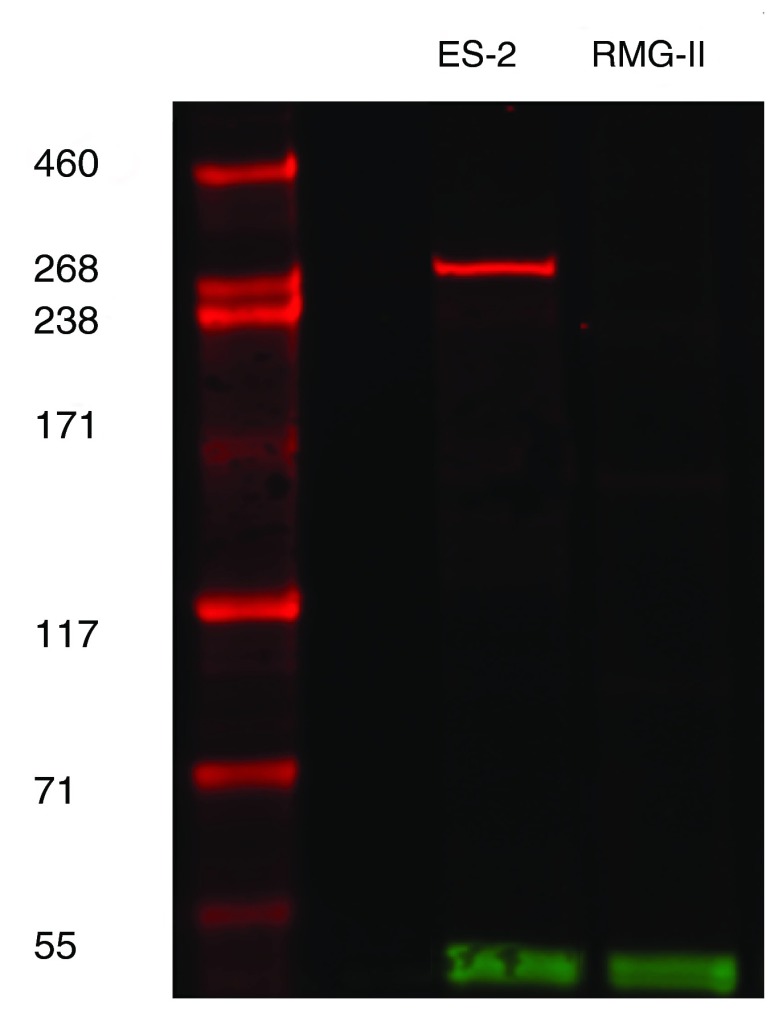
Western blot of ES2 and RMG-II clear cell carcinoma cells lines. ARID1A ( **red band**) can be seen to be present at approximately 270kD in ES2 cell line only. GAPDH at 37kD ( **green band**) represents loading control.

To determine the correct cell line to utilise and to confirm the equivocal Western blot data from Human Protein Atlas, the antibody was used to stain a Western blot of two cell lines; ES-2 and RMG-II, both of which are cell lines derived from clear cell carcinoma and have been previously demonstrated as ARID1a wild-type and mutated, respectively ^9^. It could be demonstrated that the HPA ARID1A antibody showed positive expression in ES-2 cell lines at the expected size of 270 kDa and no staining for RMG-II. The loading control of GAPDH showed that there were no loading issues ( Figure 1). Thus, these cell lines were chosen to be grown, formalin fixed and processed into paraffin wax for immunohistochemical validation.

**Figure 2.**
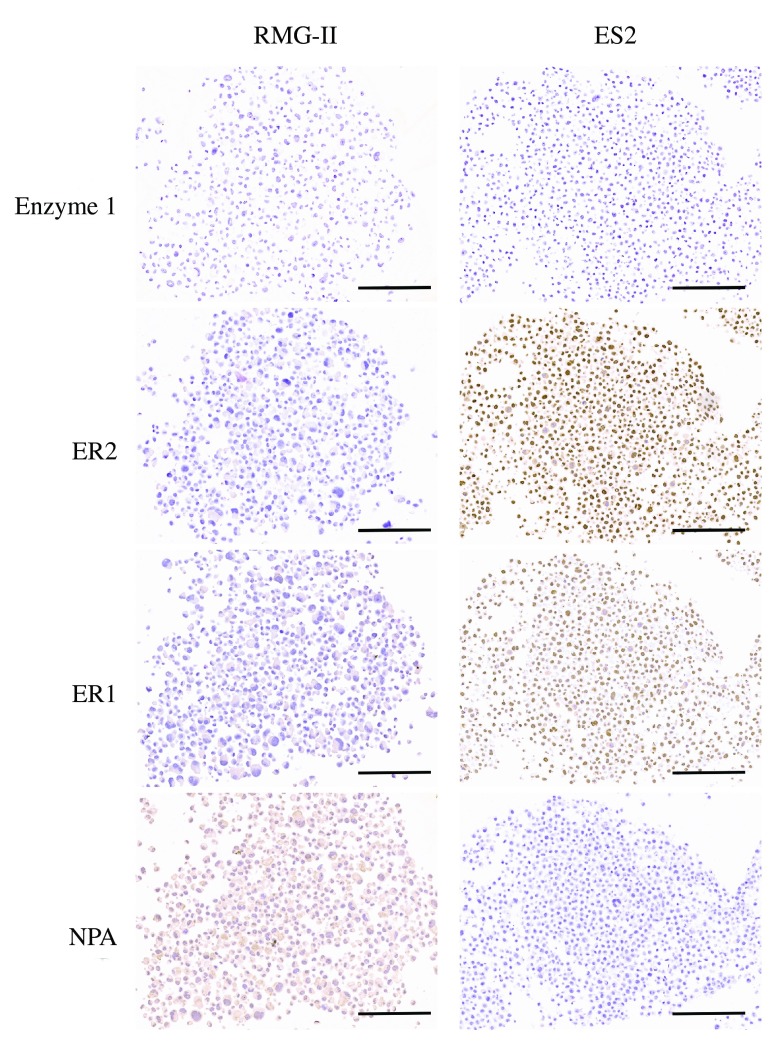
ES2 and RMG-II cell lines stained by immunohistochemistry with anti-ARID1A using three antigen retrieval conditions, ER1, ER2 and Enzyme 1. NPA denotes No Primary antibody control and represents the ER2 condition. Bar = 200 µm.

For immunohistochemical validation, ES-2 and RMG-II cell lines were stained using three antigen retrieval conditions; ER1 (Sodium Citrate, pH6), ER2 (Tris/EDTA, pH9) and Enzyme 1 (Proteinase K, 100 µg/ml) at a fixed antibody concentration of 1 µg/ml. The enzyme retrieval demonstrated no nuclear signal for either ES2 or RMG-II cell pellets and was discarded for future work ( Figure 2; Dataset a). The ER2 condition did demonstrate significant nuclear staining in the ES2 cell pellet with minimal background staining in the RMG-II cell pellet ( Figure 2; Dataset b). However, the staining in the ER1 condition was determined to give the best signal:noise ratio with no background cytoplasmic staining and crisp nuclear staining for the cell pellet ( Figure 2; Dataset c). Control slides, omitting the primary antibody, were negative except for the ER2 condition in the RMG-II cell pellet where a weak cytoplasmic background could be seen ( Figure 2; Dataset d). Thus there was minimal background inherent in the staining procedure. It was therefore determined that the antibody showed specificity for formalin-fixed paraffin embedded tissues and could be run on murine tissue.

**Figure 3.**
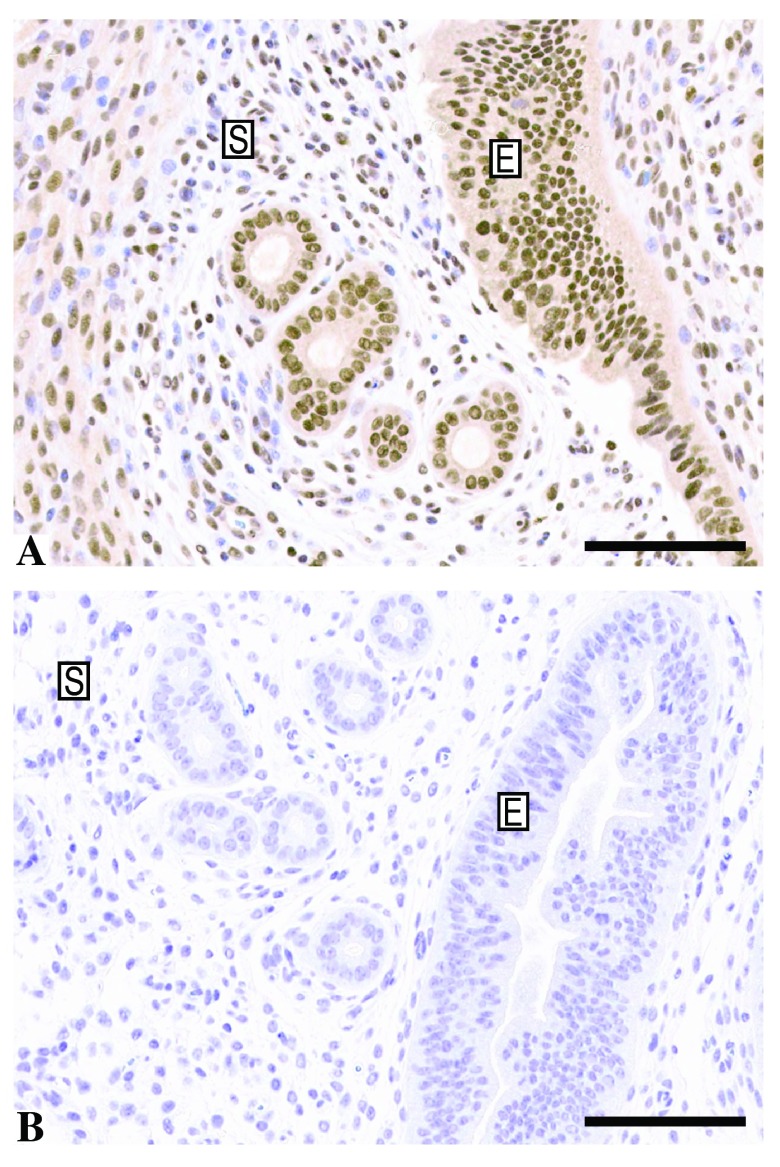
**A**) Murine uterine tissue stained by immunohistochemistry with anti-ARID1A using the ER1 condition and a concentration of 1 ug/ml demonstrating clear nuclear staining of the epithelial compartment ( **E**) and negative nuclei in the stromal compartment ( **S**). **B**) No primary antibody control of a similar area of epithelium/stroma. Bar = 100 µm.

Murine uterine tissue was used as positive control tissue samples, given the literature data on cell lines and endometrial tissue. The ER1 condition at 1 µg/ml demonstrated clean nuclear staining in the uterine epithelial compartment as well as nuclear staining of stromal cells. However, the nuclear staining in the stroma was not universal and distinct negative nuclei could be seen ( Figure 3A; Dataset e). There was no cytoplasmic or extracellular stromal background staining present and the antibody titrated successfully losing the intensity of staining, as expected ( Dataset e). Following this, a concentration of 0.5 µg/ml was used for future preparations which provided clear and consistent staining in repeated batches using a different antibody lot ( Dataset f). A No Primary antibody control (NPA) showed no staining in the epithelial or nuclear compartment ( Figure 3B; Dataset e).

**Figure 4.**
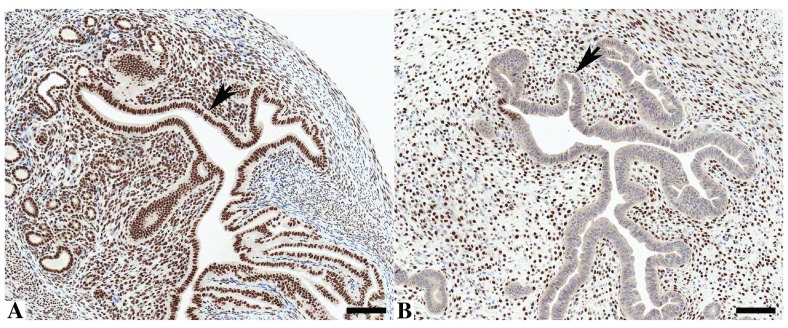
Murine uterine tissue stained by immunohistochemistry with anti-ARID1A demonstrating nuclear staining in wild-type mice ( **A**) but loss of epithelial staining after ARID1A knock-out in *Arid1a ^fl/fl^* mice ( **B**). Bar = 100 μm.

Finally, when applied to a genetically engineered, tamoxifen-induced, Arid1a knockout mouse model, the staining in the uterine epithelium could be almost completely abrogated (Arrow, Figure 4b) when compared to the same area in a wild-type animal (Arrow, Figure 4a) with a small focal area of epithelial staining still present. There was no effect of the KO on the staining in the stromal compartment.

Whole slide images from antibody validation of HPA005456 for immunohistochemistry - Version 2 Detailed legends for each dataset (Datasets a–f) can be found in the text file provided. For Version 2 an additional image was added to Dataset e.

## Conclusions

It is clear from the use of ES2 and RMG-II cell lines that the Atlas Antibodies ARID1A antibody is specific for ARID1A in both Western blots and formalin-fixed paraffin embedded preparations of human origin and, coupled with the literature evidence, that it is validated in human tissue.

The staining pattern when applied to murine uterus showing a clear nuclear pattern, where there is a high level of sequence homology between the two species, is again consistent with the literature on this protein. When stained on an ARID1a knockout mouse model, the staining could be almost completely abrogated in the epithelial compartment but not in the stroma. Knockout mice generated in this manner are almost never 100% complete as in some cells recombination will not be induced due to issues such as low ligand (Tamoxifen) penetration or failure of the ligand to induce recombination, thus explaining the small focal area of epithelial staining. The difference in staining in the two compartments is also likely related to the same effect, as all other controls, such as omission of primary antibody remained negative. Thus, given the overwhelming data from other sources, it is likely the stromal staining reflects continuing Arid1a expression in this specific model system.

Therefore, in conclusion when taken in combination, it is clear that the anti-human ARID1a antibody is cross-reactive with murine tissue and can be used for this purpose.

## Data availability

F1000Research: Dataset 1. Whole slide images from antibody validation of HPA005456 for immunohistochemistry - Version 2, 10.5256/f1000research.5514.d41579 ^10^
