# Supplementary material for: Application of ARID1A to murine formalin-fixed paraffin embedded tissue using immunohistochemistry
Source: F1000Res. 2015 Jan 6;3:244. Originally published 2014 Oct 15. [Version 2] doi: 10.12688/f1000research.5514.2 (PMC4304311; doi:10.12688/f1000research.5514.2)
Supplement: Peer review history

# Peer review: Referee response for version 2

**DOI:** 10.5256/f1000research.6423.r7236

**Published:** 2015-01-12

**Authors:** Stephen McQuaid

**Affiliations:** 1 Center for Cancer Research and Cell Biology, Queen's University Belfast, Belfast, UK

All concerns have been addressed.

I have read this submission. I believe that I have an appropriate level of expertise to confirm that it is of an acceptable scientific standard.

# Peer review: Referee response for version 2

**DOI:** 10.5256/f1000research.6423.r7235

**Published:** 2015-01-09

**Authors:** Andrew Chalmers

**Affiliations:** 1 Department of Biology and Biochemistry, University of Bath, Bath, UK

The authors have addressed my comments/concerns satisfactorily and I am happy to approve the manuscript.

I have read this submission. I believe that I have an appropriate level of expertise to confirm that it is of an acceptable scientific standard.

# Peer review: Referee response for version 1

**DOI:** 10.5256/f1000research.5886.r6848

**Published:** 2014-12-02

**Authors:** Stephen McQuaid

**Affiliations:** 1 Center for Cancer Research and Cell Biology, Queen's University Belfast, Belfast, UK

This article, by Howatt *et al. *validates an antibody to ARID1A in murine FFPE samples, is a good example of the correct validations procedures that must be met when validating an antibody for use in tissue sections. Issues are relatively minor:

1. What was the rationale for a starting concentration of 1μg/ml for initial validations?
2. There is a focal region of the KO epithelium which clearly appears to be positive. The authors would need to explain this.
3. Can the authors please add more discussion of the nature of the staining of the stromal cells. Do they consider this to be non-specific? Is such staining of stroma present in human tissue sections?
4. Control slides, omitting primary antibody, were carried out during the cell line phase of the experiments. Were such slides run on the murine tissue sections and can the results be included in the data. I note that the stromal staining was still present at selected concentration of 0.5μg/ml (dataset f)

I have read this submission. I believe that I have an appropriate level of expertise to confirm that it is of an acceptable scientific standard, however I have significant reservations, as outlined above.

# Response

**Published:** 2014-12-31

**Authors:** Will Howat

**Affiliations:** Cancer Research UK Cambridge Institute, UK

Dear Dr McQuaid,

Thank you for your comments which we agree with and have taken on board. Specifically:

- All of the validations conducted at the Histopathology/ISH facility start with a dilution of 1:100 from the manufacturers provided stock concentration and we perform 3 retrieval methods; Sodium Citrate pH6, Tris EDTA pH9 & Proteinase K. We titrate as appropriate following establishment of the optimal retrieval method. The manufacturers original stock concentration was 100ug/ml, hence the 1ug/ml starting concentration. They have subsequently changed their stock concentration to 200ug/ml.
- We believe that the knockout model recombining a floxed Arid1a with CRE-ERT2 is not 100% complete as in some cells recombination will not be induced due to issues such as low ligand (tamoxifen) penetration or failure of the ligand to induce recombination. Thus explaining the area of focal staining in the epithelium. Similarly, although we have not investigated this further, we believe that the same problem underlies the stromal staining and is likely due to penetrance of the tamoxifen after being delivered intraperitoneally. Thus, given the wealth of data and the no primary antibody controls, now included in Fig 3, we feel that the stromal staining is not non-specific. Stromal staining does indeed occur in human tissue.

All of the above have now been included in the conclusion section of the article.

Kind Regards,

Will Howat

# Peer review: Referee response for version 1

**DOI:** 10.5256/f1000research.5886.r6651

**Published:** 2014-11-21

**Authors:** Andrew Chalmers

**Affiliations:** 1 Department of Biology and Biochemistry, University of Bath, Bath, UK

The manuscript by Will Howat and colleagues validates an anti-ARID1A antibody and provides a good example of an antibody validation paper. In particular the use of negative cell lines and knockout mouse tissue demonstrate the extent of specificity of the antibody. The data is mostly well presented and clearly explained in the text.

The manuscript would be suitable for indexing if some, mostly minor, issues can be addressed.

1. I think the title should mention “antibody” to make clear it’s an antibody that is being validated.
2. The authors say there is 95% sequence homology between human and mouse, it would be interesting to see how conserved the sequence the antibody was raised against is.
3. The Materials and Methods give a good overview of the reagents and methods used for the IHC, but there is little information about the western blotting. The reagents and methods used should be added.
4. It would be good to cite the source of the cells used and the mouse tissue.
5. I was not clear if the lack of staining in RMG-II cells was expected or an unexpected but useful result? This should be explained.
6. Figure 1 legend. Should read “represents *the* loading control”
7. In figure 4B the KO epithelium is clearly negative, except one region in the top left which looks positive, I wondered what the authors felt about this?
8. It is interesting that the stromal staining appears to be non-specific, I wonder if the authors ever did a no primary control? Does the stroma still stain? Also it would be worth mentioning this staining in the conclusions.
9. Author contributions. Should it read “conceived the idea behind the article” or similar wording ?

I have read this submission. I believe that I have an appropriate level of expertise to confirm that it is of an acceptable scientific standard, however I have significant reservations, as outlined above.

# Response

**Published:** 2014-12-31

**Authors:** Will Howat

**Affiliations:** Cancer Research UK Cambridge Institute, UK

Dear Dr Chalmers,

Many thanks for your comments which we agree with and have taken on board. Specifically:

- We have modified the title to read "Application of anti-ARID1a antibody..."
- We have BLAST searched the protein sequence and it confirms 95% homology for the sequence used for immunisations. This has been added to the methods.
- We accept that the methods detailing the Western blotting are more limited than the IHC methods, as is the case of the methods detailing the production of the knockout mouse model. However, we feel that the article was designed as an example of antibody validation for immunohistochemistry and that while western blotting provides important additional data, it is not the focus of the article. We thus feel that there is sufficient information in the details behind the western blotting to repeat the experiment, without clouding the article with the full methods.
- The sources of the cells and mouse tissue have now been cited.
- The lack of staining of RMG-II cells was consistent with the literature (Anglesio *et al.*) and this information has been added to the results section.
- The figure legend has been modified.
- We believe that the focal staining is a result of incomplete recombination floxed Arid1a allele and Cre/ERT2. The resulting knockout is almost never 100% complete as in some cells recombination will not be induced due to issues such as low ligand (tamoxifen) penetration or failure of the ligand to induce recombination. Similarly, we believe that the staining in the stromal compartment is specific but represents a failure of delivery of Tamoxifen or of recombination. The data from no primary antibody (NPA) control which are all completely clean, now included in Fig 3, helps to demonstrate this. We have included these points in the conclusion.
- The author contributions have been modified.

Kind Regards,

Will Howat
